# Detrimental effects of excessive fatty acid secretion on female sperm storage in chickens

**DOI:** 10.1186/s40104-020-0432-8

**Published:** 2020-04-02

**Authors:** Chaoliang Wen, Chunning Mai, Bin Wang, Junying Li, Congjiao Sun, Ning Yang

**Affiliations:** 1 Department of Animal Genetics, Breeding and Reproduction, College of Animal Science and Technology, China Agricultural University, Beijing, 100193 China; 2 National Engineering Laboratory for Animal Breeding and Key Laboratory of Animal Genetics, Breeding and Reproduction, Ministry of Agriculture and Rural Affairs, China Agricultural University, Beijing, 100193 China

**Keywords:** Chickens, Female sperm storage, Lipid peroxidation, Metabolomics, Sperm storage tubules

## Abstract

**Background:**

Female sperm storage (FSS), the maintenance of sperm inside the female reproductive tract for an extended period of time, is pervasive among organisms with internal fertilization. Because FSS enables asynchronous mating and fertilization, it could be extremely important to reproduction. However, the physiological mechanisms underlying prolonged preservation and maintenance are poorly understood. Here, we used chicken, a typical oviparous animal, to determine the mechanisms ensuring sperm functionality in sperm storage tubules (SSTs).

**Results:**

We performed an insemination experiment on over two thousand hens at two periods, and found that the FSS capabilities varied widely among individuals. Except for the differences in the SST density between the two groups with distinct FSS abilities, we quantitatively profiled small-molecule metabolites derived from SST cells, and identified 28 metabolites with differential expression. In particular, high levels of lipids, fatty acids and lipid peroxidation product were observed in hens with low FSS capability. Pathway analysis showed that these differential metabolites were significantly enriched in the biosynthesis of unsaturated fatty acids. Moreover, we detected the total antioxidant capacity and lipid peroxidation level of SSTs, and found that chickens with a lower FSS ability had a significantly higher content of lipid peroxidation end-product, which was 2.4-fold greater than chickens with a higher FSS capability, and no significant difference was found in the total antioxidant capacity between these two groups.

**Conclusions:**

Our findings reveal that the long-term storage of sperm and the maintenance of their function in the female reproductive tract require an adequate microenvironment. The superabundance of fatty acids secreted by SST cells had detrimental effects on sperm storage in the female reproductive tract. Lipid peroxidation produces toxic biological substances that may cause irreversible damage to resident spermatozoa, resulting in short-term sperm retention and decreased fertility. Our findings provide new avenues for studying sperm storage and sustaining fertility.

## Background

For internally fertilizing animals, female sperm storage (FSS) is an essential process in reproduction because it enables mating and fertilization to be asynchronous [1, 2]. Sperm storage is most commonly defined as the extended maintenance of viable sperm within the female reproductive tract (RT) [3]. In animals as diverse as mammals and insects, females possess specialized morphological structures for sperm storage; these structures are usually associated with the epithelium of the RT lumen or in blind-ended structures that maintain sperm viability until they are used for fertilization [4, 5]. The duration that sperm remain in storage varies greatly among different organisms. Dogs can store fertile spermatozoa for 9 days [6], and some turtles can sustain sperm fertility for an impressive four years [7]. Perhaps the most remarkable duration of sperm storage is observed in ants [8] and bees [9], which can store sperm for nearly their entire lives. However, attempts to maintain spermatozoa function at ambient or body temperature in the laboratory typically reach their limit after 2~3 days. If we could elucidate and characterize the mechanism behind this process, thereby promoting prolonged sperm storage at ambient temperatures, the practical benefits would be enormous, especially in genetic resource conservation, sperm separation and artificial insemination.

As typical oviparous animals, avian species are suitable experimental models for investigating the potential mechanisms of FSS because it is easy for us to assess the duration of sperm storage by a series of fertile eggs following artificial insemination. The main residence location of spermatozoa in the avian oviduct is termed sperm storage tubules (SSTs), which are located in the internal mucosal folds of the utero-vaginal junction (UVJ). Sperm can be stored and can survive here for a few days to several weeks after copulation or insemination [10–12]. Numerous traditional anatomic and histological studies have consistently reported that SSTs were lined by a single layer of non-ciliated cells that were formed by the invagination and differentiation of mucosal surface epithelium [13–17]. Despite several comparative studies among species or breeds has been suggested that the sperm storage capacity and duration of storage are related to the relative number of SSTs present in the RT [18, 19]. From a functional standpoint, the capacity of females to store spermatozoa in SSTs for long periods requires an adequate biochemical environment to sustain both the viability and fertilizing potential of spermatozoa. Significantly, a considerable body of evidence has suggested that sperm motility is amenable to upregulation or downregulation by substances derived from the female RT [4, 9, 20–24].

In the past decades years, secretion of avian SSTs has been studied extensively through histochemical approaches. Fujii [25] described large amounts of lipid components present in the perinuclear region of SST cells, and similar results were reported by Friess et al. [26], Schuppin et al. [27] Bakst [28] and Huang et al. [29]; however, the distinct role of lipids remains to be established. It should be noted that sperm stored in SSTs require sufficient substances for energy metabolism to maintain sperm survival over prolonged periods. Bakst et al. [30] and Huang et al. [29] assumed that lipids may be hydrolyzed in SST cells and released as fatty acids into the SST lumen. Then, sperm uptake and metabolize those fatty acids to extend their viability and activity. However, sperm metabolize carbohydrates (such as glucose and fructose) more generally under aerobic or anaerobic conditions, at least *in vitro* storage [31, 32]. An early histological staining study in chickens [28] showed that the cytoplasm of SST cells contains glycogen. In addition, lipids are among the major components of sperm membranes involved in a series of processes that ultimately influence their fertilizing ability. As a consequence, a highly efficient antioxidant system must be present to prevent or repair sperm membrane damage caused by peroxidation during storage [5, 33]. Most literatures have hypothesized that the lipids present in SSTs may participate in the defense against peroxidation [33, 34].

Previous observations in seminal plasma revealed the presence of a complex antioxidant system involving water-soluble (such as ascorbic acid, glutathione and uric acid) and lipid-soluble antioxidants (such as vitamin E) in this fluid [33, 35, 36]. However, the antioxidant system present in SSTs has been poorly studied. Metabolomics as a global chemical phenotyping method is now more widely available and has become a new approach for facilitating the understanding of the mechanisms of biological and biochemical processes in complex biological systems. Therefore, we performed untargeted metabolomics to characterize the metabolic profiling of SSTs in chickens, and to identify some small molecules that significantly differ between the hens with a capacity for the long- and short-term storage of sperm. The most important objectives of this study were to uncover the physiological mechanisms that enable prolonged sperm storage.

## Methods

### Animals and FSS ability measurement

A pedigreed chicken line from Wen’s Nanfang Poultry Breeding, Co., Ltd. (Guangdong, China) was used in this study. The breed had been previously selected based on laying intensity and persistency but not on the duration of fertility or related characteristics. Chickens were housed identically in individual cages and properly identified by their cage number. They were free access to feed and water and kept under a 16 h light: 8 h dark photoperiod. All hens (*n* = 2202) were artificially inseminated once in the afternoon on two consecutive days with 50 μL of diluted pooled semen at 35 weeks of age. To reduce individual male effects, semen concentrations and motility of each rooster were evaluated by visual observation with a light microscope. Males that have a lower or higher semen quality were discarded. The remaining roosters were employed for the insemination experiment. Ejaculates from five males were pooled and diluted 1:1 with a glutamate diluent [37] so that approximately equal numbers of spermatozoa were inseminated into the oviduct at each insemination [10, 38].

Eggs were collected and marked daily from the day after the final insemination for 12 days (period 1, from 245 to 256 days of age). All eggs were candled on day 18 of incubation. Based on the incubation results, the individual fertility (FE) and fertility duration days (FDDs) were calculated using a customized R script. FE was calculated as the ratio of the total number of fertile eggs to the hatching egg production (HEP, total number of eggs incubated). FDD was defined as the number of days from the day after insemination to the last fertile egg before two cumulative infertile eggs [10]. This experiment was replicated at 54 weeks of age, and eggs were collected for 15 days (period 2, from 378 to 392 days of age) because the laying rate was decreased with the aging process.

### Genetics parameter estimation

The heritability (*h*^*2*^) for FE and FDD at the two periods together with phenotypic and genetic correlations were estimated using ASReml based on animal model. Hens with high egg production offer more information on fertilization than hens with low egg production [10]. To minimize the errors caused by a fewer number of eggs incubated, the corresponding FE and FDD were regarded as missing values, if the hens had a HEP less than four in period 1 or less than five in period 2.

### Tissue preparation and histologic estimation for the SST density

To further ensure high-quality phenotypic data, we removed the birds with HEP less than eight in any of the two periods. Subsequently, we selected one hundred hens from the remainder chickens (*n* = 1585) and divided them into the higher and lower sperm storage ability groups (HFSS and LFSS, respevtively) based on the FE and FDD during the two experimental periods. Hens with FDD ≥ 11 and FE > 90% in both periods were considered the HFSS group, while hens with FDD ≤ 10 and FE < 90% were viewed as the LFSS group. Body weight was measured with an electronic scale (to the nearest 5 g), and all hens were then euthanized by cervical dislocation at 48 h after insemination. The UVJ containing the SST was isolated immediately after opening the abdomen according to Bakst et al. [16], being longitudinally divided into two equal pieces. One piece of the UVJ tissue was fixed in 10% neutral buffered formalin for subsequent histological examination. The UVJ mucosa of the remaining tissues was scraped with a scalpel [39] and snap-frozen in liquid nitrogen, prior to storage at − 80 °C until further processing.

After fixation, dehydration and clearing, the UVJ samples were embedded in paraffin wax, and transversely sectioned at 4 μm thick. Sections were then stained with hematoxylin and eosin. For image analysis of SST density in the UVJ mucosal folds, sections were examined under a light microscope with computer-assisted software (Image-Pro plus 6.0). The percentage of SST area relative to the entire examined area in the mucosa was determined in more than four mucosal folds of UVJ based on a previously reported method [29]. One-way ANOVA was used to investigate the difference in the ratio of SST area in the UVJ mucosa between the two groups by the aov function in the R program (https://www.r-project.org/). The difference was considered to be statistically significant if the *P* was less than 0.05.

### Metabolomics analysis

#### Sample preparation

To explore the differences in endogenous small molecule of SSTs between HFSS and LFSS groups, eight birds per group were further selected for untargeted metabolic profiling analysis. The selection criteria were a) the FE and FDD between two groups were extremely different in both periods; b) the body weight and SST density were no significantly different; c) the hens were from different pedigrees. The eligible UVJ mucosal tissues were homogenized under liquid nitrogen. The homogenized samples (200 mg) were further powdered by the high flux organization grinding apparatus and ultrasonic machine. Sample preparation was conducted using an aqueous methanol (1:4) extraction process to remove the protein fraction while allowing for maximum recovery of small molecules [40]. After centrifugation, each supernatant was frozen, vacuum dried, and divided into two fractions: one for analysis by ultrahigh-performance liquid chromatography mass spectrometry (UPLC-MS) and one for gas chromatography mass spectroscopy (GC-MS).

#### UPLC-MS assay

The sample extract destined for UPLC-MS analysis was dissolved in 400 μL of aqueous methanol (1:1). The mixture was filtered through a 0.22-μm membrane and detected with a Waters ACQUITY UPLC system (Waters, Milford, MA, USA) using an ACQUITY UPLC HSS T3 column (150 mm × 2.1 mm, 1.8 μm) and a Thermo LTQ-Orbitrap XL mass spectrometer (Thermo Fisher Scientific, Waltham, MA, USA) equipped with an electrospray ionization source.

#### GC-MS assay

Heptadecanoic acid was used as an internal quantitative standard. The derivatization protocol was followed according to a previous study [41]. The resulting sample was subjected to GC-MS (Agilent 7890A/5975C) using an HP-5MS capillary column (5% phenylmethylsiloxane: 30 m × 250 μm, 0.25 μm; Agilent, J & W Scientific, Folsom, CA, USA).

#### Data extraction and metabolite identification

To minimize system errors, we introduced quality control samples [42]. The original UPLC-MS and GC-MS data were separately converted to the mzXML and netCDF formats, and each set of converted data was preprocessed in the R program by the XCMS package (v3.1.3) to obtain a table of time-aligned detected features with their retention time, mass-to-charge ratio and intensity in each sample. The mass spectra features from the UPLC-MS analysis were matched to known metabolites in the Human Metabolome Database, METLIN, and MassBank of North America. The metabolite annotation of the GC-MS data was performed with an Automatic Mass Spectral Deconvolution and Identification System, referenced to the databases of the National Institute of Standards and Technology and the Wiley Registry of Mass Spectral Data.

#### Statistical analysis

The batch normalization process was employed for the UPLC-MS data, and the GC-MS dataset was normalized to the internal standard. The normalized dataset was then imported into the SIMCA-P (v13.0) for multivariate statistical analysis. Prior to multivariate analysis, the resulting data matrices were mean-centered and scaled to unit variance. The unsupervised principal component analysis (PCA) was conducted to assess the separability of the samples. The differentially expressed metabolites (DEMs) between the HFSS and LFSS were identified and evaluated by one-way ANOVA and variable importance in projection (VIP, deriving from the orthogonal projections to latent structures discriminant analysis) values. The metabolites with both multivariate and univariate statistical significance (VIP > 1 and *P* < 0.05) were considered DEMs. The Pearson correlation coefficient was calculated with the psych package in the R program for assessment of the metabolite correlation, and the *P* values were adjusted for multiple testing using the Benjamini-Hochberg method. Moreover, the metabolites were subjected to MetaboAnalyst (V3.0, https://www.metaboanalyst.ca/) for metabolic pathway analysis [43].

### Determination of the total antioxidant capacity and malondialdehyde content

The total antioxidant capacity (T-AOC) of UVJ musical tissues was measured with the T-AOC detection kit (Beyotime, China) with a rapid 2,2′-azino-bis (3-ethylbenzthiazoline-6-sulfonic acid) method. Approximately 20 mg tissue samples were homogenized on ice with 100 μL of PBS. The homogenates were incubated at 4 °C for 10 min and centrifuged at 12,000 r/min for 5 min at 4 °C. The supernatants were collected, and the experiments were conducted according to the manufacturer’s instructions. The absorbance of the soluble end product was detected at 414 nm using a fluorescence microplate reader (Molecular Devices, USA). T-AOC was calculated according to the standard curve constructed with a Trolox standard solution (a water-soluble analog of vitamin E).

The lipid peroxidation level was detected by an malondialdehyde (MDA) assay kit (Beyotime, China) based on the chromogenic reaction of MDA and thiobarbituric acid. The absorbance of the MDA-thiobarbituric acid adduct was measured at 535 nm. The experiments were conducted according to the manufacturer’s protocol.

## Results

### Duration of sperm storage after insemination

The daily changes of laying rate and fertility for the experimental population are shown in Additional file 1: Figure S1. The average fertility was higher than 94.24% during the first eight days after insemination at period 1, after, which the fertility decreased but was maintained 72.26% at day 12. The age of hens had a major effect on egg production, which considerablly decreased in period 2 (61.84%) compared to that in period 1 (83.76%). Thus, we collected eggs for 15 days in period 2. Although a systematic decrease in fertility was observed between period 1 and period 2, the daily changed in fertility in period 2 was similar to that in period 1, which decreased sharply at day 8 and approached 22.62% on day 15.

The individual FE and FDD were used as predictors to quantify the duration of sperm storage. The descriptive statistics of HEP and sperm storage ability in the two experimental periods are summarized in Additional file 2: Table S1. A total of 1.32% and 8.98% of chickens (Additional file 3: Figure S2a) were excluded from the individual FE analysis during period 1 and period 2, respectively, considering that such hens with low laying performance did not allow accurate determination of the sperm storage ability. The range of FE and FDD among individuals varied widely (Additional file 3: Figure S2b and S2c), even though the insemination conditions were the same and optimal, especially in period 2, indicating large variations in the sperm storage ability in this population.

### Genetic parameters for FSS traits

In order to examine whether the indexes that we selected are suitable for evaluating the ability of FSS, variables measured at different ages were considered as different traits. The *h*^*2*^, and phenotypic and genetic correlations are given in Table 1. The *h*^*2*^ estimates for FE and FDD in the first experimental period were 0.12 and 0.11, respectively. For period 2, the *h*^*2*^ of FE was 0.25, and that of FDD was 0.22. The genetic correlation among these traits was 0.35 ~ 0.98, with respect to the phenotypic correlation between FE1 and FE2 being slightly lower due to the different egg collection cycle between the two experiments.

**Table 1:** Heritability (on diagonal), phenotypic (above diagonal) and genetic (below diagonal) correlations for FSS traits. Standard errors of estimates are in parentheses

Traits^a^	FE1	FDD1	FE2	FDD2
FE1	**0.121 (0.036)**	0.824	0.203	0.170
FDD1	0.963 (0.040)	**0.112 (0.036)**	0.139	0.128
FE2	0.654 (0.158)	0.374 (0.190)	**0.248 (0.055)**	0.809
FDD2	0.577 (0.171)	0.348 (0.191)	0.978 (0.020)	**0.218 (0.053)**

^a^: FE1 and FDD1 represent individual fertility and fertility duration days during the first experimental period from 245 to 256 days of age (12 days), repectively. FE2 and FDD2 represent individual fertility fertility duration days during the second experimental period from 378 to 392 days of age (15 days), respectively

### Difference in the SST density

**Fig. 1:**
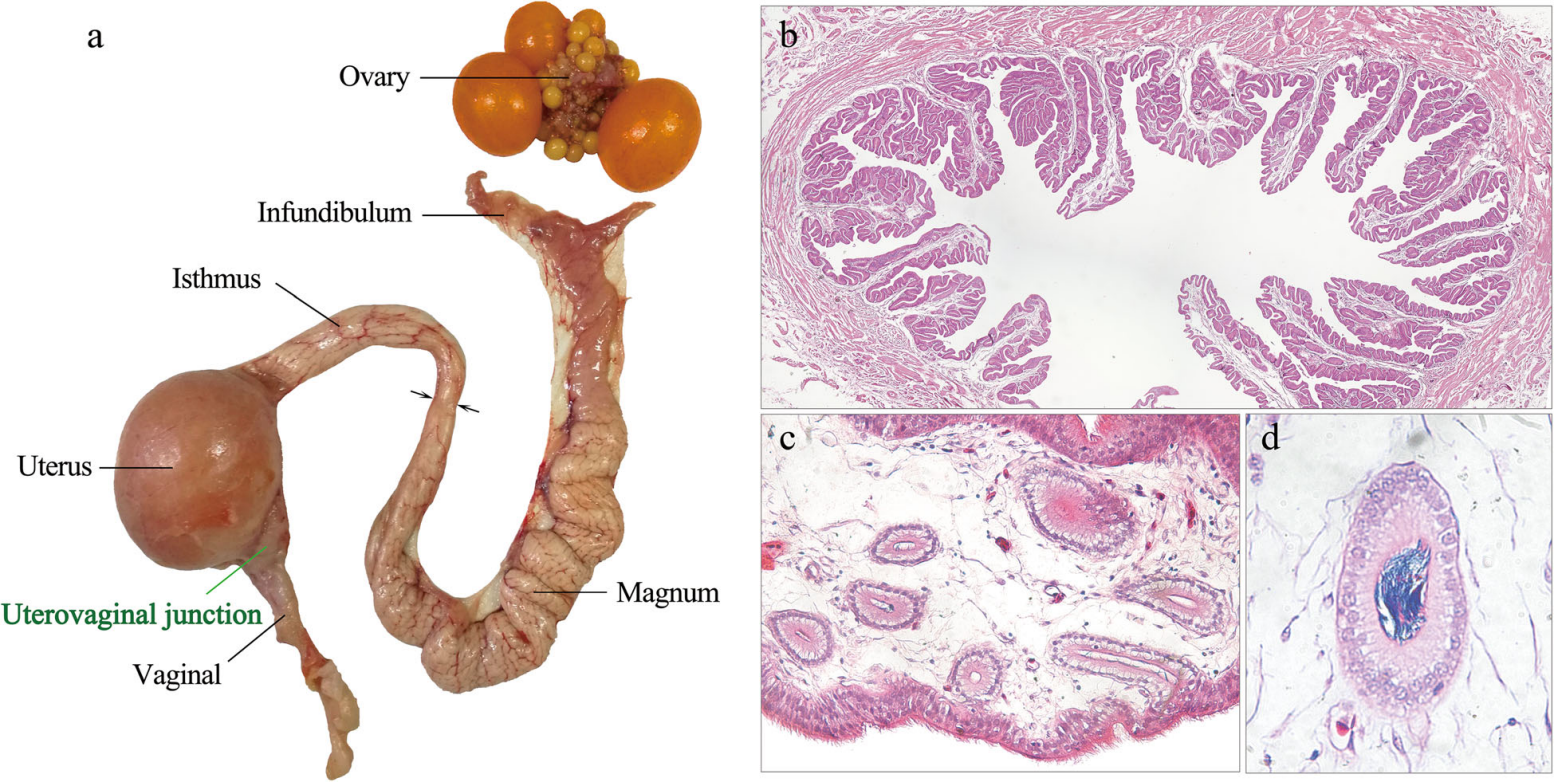
Oviduct of an adult hen with special reference to sperm storage sites. **a** Diagrammatic representation of the chicken oviduct. The main sperm storage site is located in the uterovaginal junction (UVJ). **b** A light micrograph (stained with hematoxylin and eosin, 40×) showing the mucosal folds of UVJ. **c** Transverse sections of sperm storage tubules (SSTs) in mucosal fold of UVJ (400×). **d** Sections of a single SST with sperm bundled in the lumen (1000×)

**Fig. 2:**
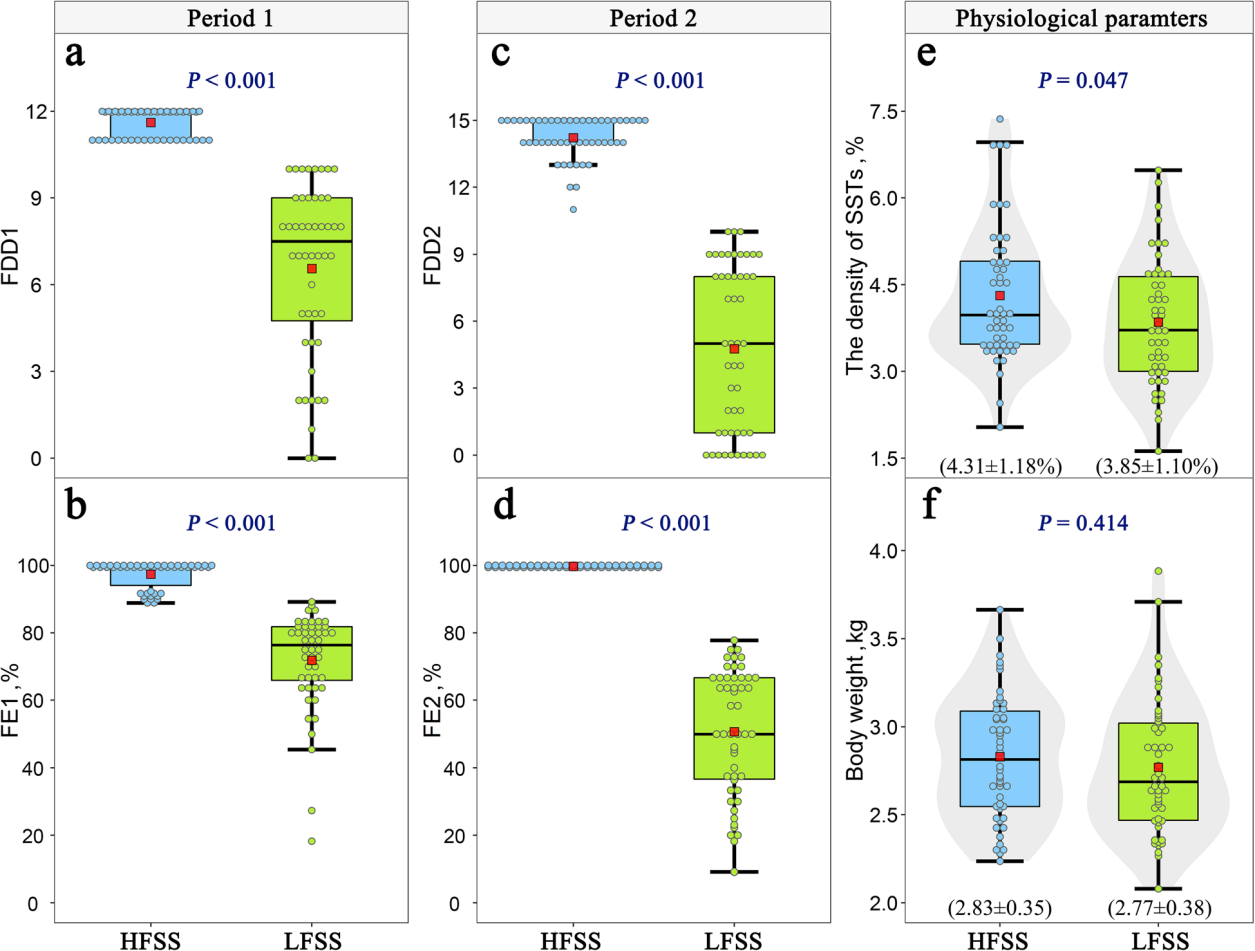
Differences in the physiological traits between the hens with higher and lower durations of female sperm storage (HFSS and LFSS). **a**-**d** The fertility duration days (FDDs) and fertility (FE) during the two-experiment periods. **e** The density of SSTs. **f** Body weight. The center red point is the mean value in the corresponding group, and for **a**-**f**, data are expressed as the mean ± SD

Given that SSTs located in the UVJ (Fig. 1a) are the main sperm storage site, we first compared the SST density in the UVJ mucosal folds (Fig. 1b) among hens with distinct sperm storage abilities. Light microscopy revealed that the SST was tubular invaginations of the UVJ surface epithelium (Fig. 1c). However, unlike the UVJ, which was lined by a pseudostratified layer of ciliated and nonciliated columnar cells, the SST epithelium was made of simple columnar cells that were nonciliated. More dispersed sperm were observed in the lumen of the SST (Fig. 1c), and the sperm bundle was sporadically observed in the hens (Fig. 1d). According to the individual FE and FDD, we selected one hundred hens and divided them into two groups (Fig. 2a-d). The ratio of the SST area in the UVJ mucosal folds was significantly higher in chickens in the HFSS group than in chickens in the LFSS group (Fig. 2e), and no significant difference in body weight was found between these two groups (Fig. 2f).

### Differential metabolites identification

**Fig. 3:**
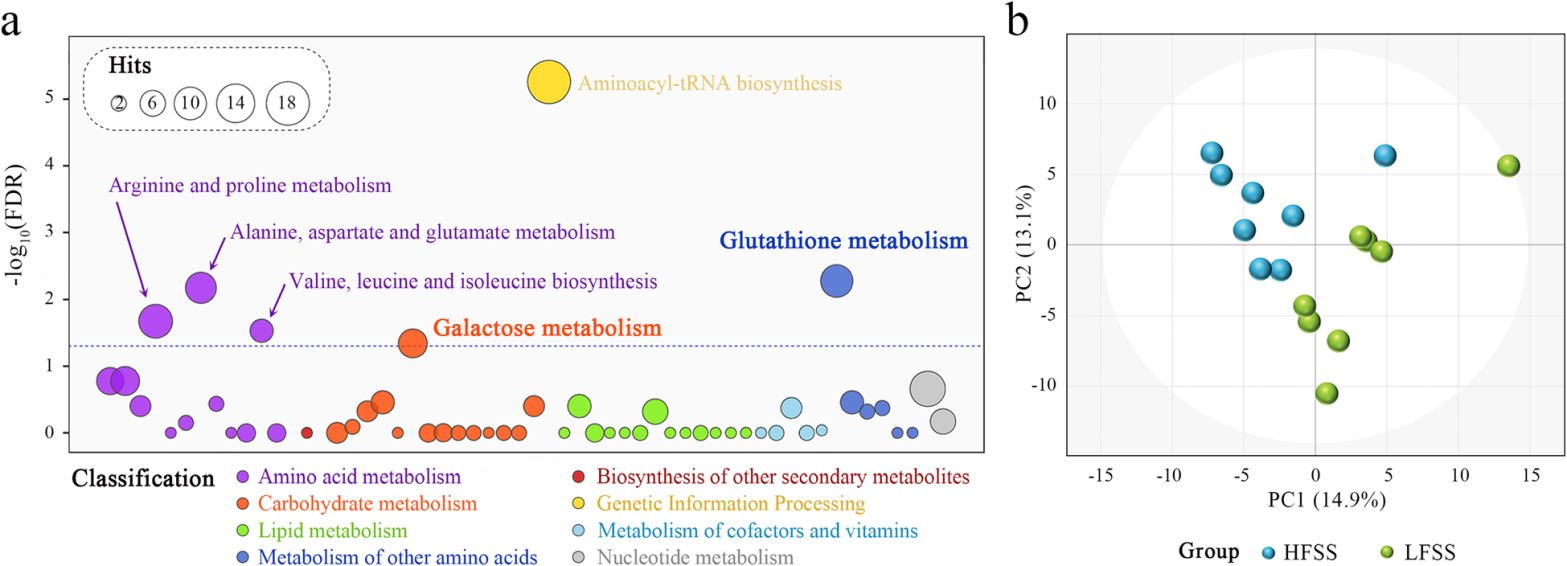
Metabolomics analysis. **a** KEGG pathway analysis for all metabolites. **b** Principal component analysis (PCA) score scatterplot of metabolic profiles. The circle represents the 95% confidence intervals of the PCA model

Apart from the difference in the SST density, the more fundamental question is how spermatozoa can durably resist the adverse effects of prolonged sperm storage in the oviduct, considering the relatively short period of sperm viability after semen collection and storage *in vitro*, but the sperm can survive for up to several weeks in the SST of the oviduct. Thus, we selected eight hens from each group for untargeted metabolic profiling analysis. The physiological and phenotypic parameters of these hens are listed in Additional file 4: Table S2. A total of 186 metabolites were obtained from the metabolic profiling of the UVJ mucosal tissues (Additional file 5: Table S3). To gain a clear understanding of the SST metabolic activity, we performed a KEGG pathway analysis of the metabolites that we identified. As expected, aminoacyl-tRNA biosynthesis and several amino acid metabolism pathway were significantly enriched; these results are unsurprising given that the metabolites we detected have many amino acids. Surprisingly, the metabolites of the UVJ mucosal sample were significantly enriched in glutathione metabolism and galactose metabolism (Fig. 3a), indicating high energy metabolic and antioxidant defense activities in the SST lumen.

**Fig. 4:**
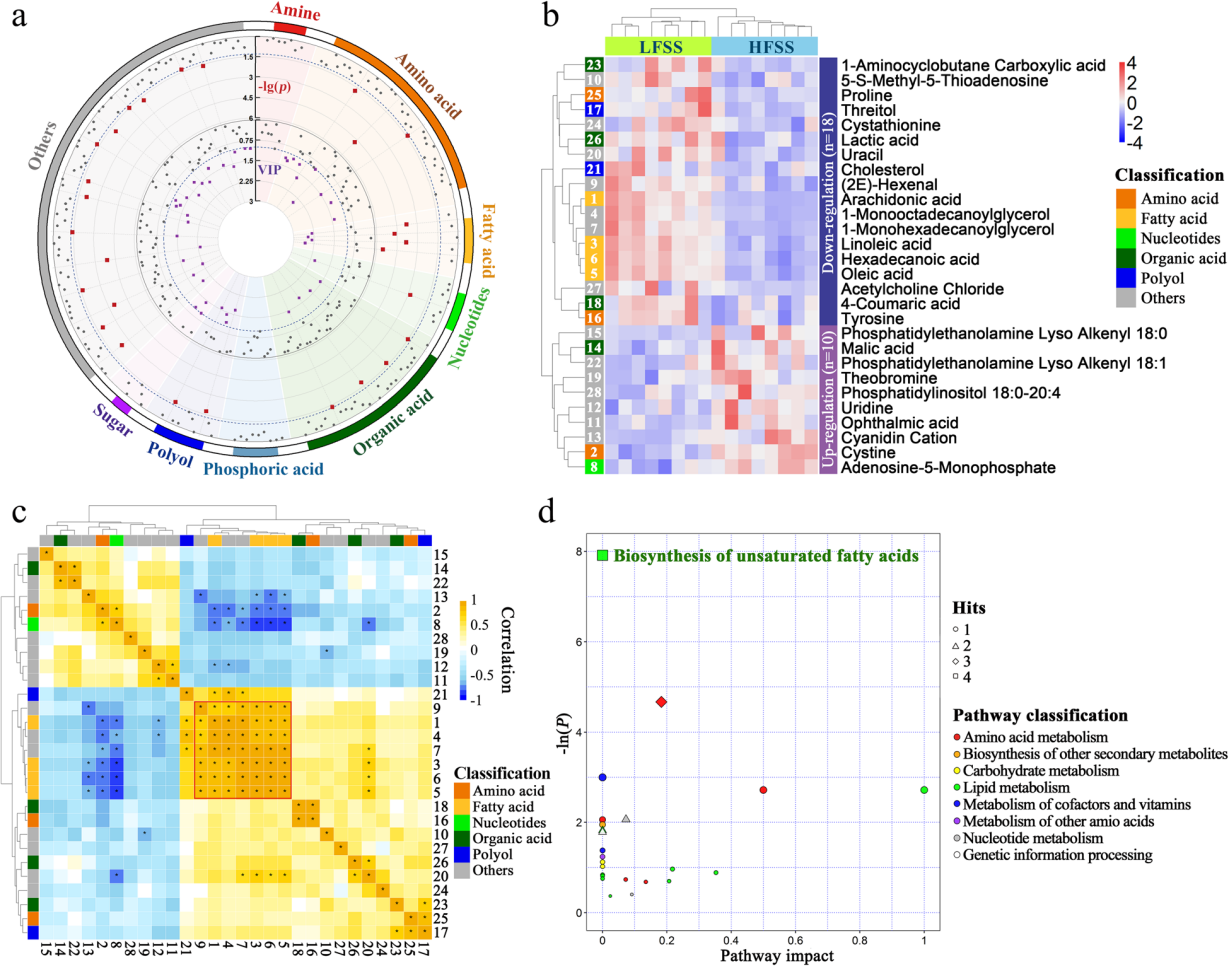
Analysis of the differential metabolites between the HFSS and LFSS groups. **a** Identification of differentially expressed metabolites (DEMs) between the HFSS and LFSS groups. The outer circle displays the *P* values of One-way ANOVA, where *P* values are plotted as −ln (*P*). The inner circle shows variable importance in projection (VIP) values of the OPLS-DA model. Each point represents a metabolite and the blue dashed line shows the significance threshold (*P* < 0.05 or VIP > 1). The gray dashed line indicates the *P* value, and the VIP of one metabolite all passed the significance threshold. **b** Heatmap of the 28 significantly different metabolites between the HFSS and LFSS hens. The heatmap is color-coded based on row z-scores. The corresponding metabolites of the Arabic numerals are soared by *P*-value. **c** Pearson correlation analysis of the 28 DEMs. The corresponding metabolites of the Arabic numerals are the same as shown in Fig. 4b and Table 2. The color of the circles indicates the magnitude of correlation between metabolites, and the symbol * indicates that a *P* < 0.05. **d** Metabolic pathway identification of DEMs. The corresponding enriched pathways are displayed in Additional file 6: Table S4

An unsupervised PCA was then conducted to visualize the differences in metabolic composition. The PCA plot showed that the two different groups were clearly separated from each other (Fig. 3b), indicating that there were visible differences in the metabolic characteristics between the HFSS and LFSS hens. The successful discrimination of the HFSS and LFSS groups led us to search for potential metabolites that may result in the differences in retaining the sperm fertilizing capacity. All of the metabolites detected by untargeted metabolomics were used for statistical analysis. A total of 28 metabolites were significantly differentially expressed between the HFSS and LFSS groups (Table 2 and Fig. 4a). As shown in Fig. 4b, 18 and 10 compounds exhibited significant decreases and increases in the HFSS set compared with those in the LFSS set, respectively.

**Table 2:** Differential metabolites in the UVJ tissue between the HFSS and LFSS hens

No.	Metabolite	Formula	HMDB ID	*P*-value^a^	*q*-value^b^	VIP^c^	FC^d^
1	Arachidonic acid	C_20_H_32_O_2_	HMDB0001043	2.89E-06	5.37E-04	2.57	0.40 ↓
2	Cystine	C_6_H_12_N_2_O_4_S_2_	HMDB0000192	1.90E-05	1.23E-03	2.47	1.17 ↑
3	Linoleic acid	C_18_H_32_O_2_	HMDB0000673	1.99E-05	1.23E-03	2.47	0.72 ↓
4	1-Monooctadecanoylglycerol	C_20_H_40_O_4_	–	1.33E-04	5.11E-03	2.33	0.61 ↓
5	Oleic acid	C_18_H_34_O_2_	HMDB0000207	1.42E-04	5.11E-03	2.33	0.70 ↓
6	Hexadecanoic acid	C_16_H_32_O_2_	HMDB0000220	1.65E-04	5.11E-03	2.31	0.75 ↓
7	1-Monohexadecanoylglycerol	C_19_H_38_O_4_	HMDB0011564	3.63E-04	9.65E-03	2.24	0.71 ↓
8	Adenosine-5-monophosphate	C_10_H_14_N_5_O_7_P	HMDB0000045	1.52E-03	3.54E-02	2.08	1.47 ↑
9	(2E)-hexenal	C_6_H_10_O	HMDB0031496	4.57E-03	9.44E-02	1.92	0.68 ↓
10	5-S-methyl-5-thioadenosine	C_11_H_15_N_5_O_3_S	HMDB0001173	8.11E-03	1.51E-01	1.83	0.86 ↓
11	Ophthalmic acid	C_11_H_19_N_3_O_6_	HMDB0005765	1.21E-02	1.87E-01	1.75	2.30 ↑
12	Uridine	C_9_H_12_N_2_O_6_	HMDB0000296	1.22E-02	1.87E-01	1.75	1.30 ↑
13	Cyanidin cation	C_15_H_11_ClO_6_	HMDB0002708	1.31E-02	1.87E-01	1.74	1.64 ↑
14	Malic acid	C_4_H_6_O_5_	HMDB0000744	1.57E-02	1.96E-01	1.70	1.26 ↑
15	Phosphatidylethanolamine lyso alkenyl 18:0	C_23_H_48_NO_6_P	–	1.59E-02	1.96E-01	1.70	2.12 ↑
16	Tyrosine	C_9_H_11_NO_3_	HMDB0000158	1.69E-02	1.96E-01	1.68	0.85 ↓
17	Threitol	C_4_H_10_O_4_	HMDB0004136	1.83E-02	2.00E-01	1.67	0.59 ↓
18	4-coumaric acid	C_9_H_8_O_3_	HMDB0002035	2.18E-02	2.25E-01	1.63	0.82 ↓
19	Theobromine	C_7_H_8_N_4_O_2_	HMDB0002825	2.33E-02	2.25E-01	1.61	1.31 ↑
20	Uracil	C_4_H_4_N_2_O_2_	HMDB0000300	2.41E-02	2.25E-01	1.61	0.66 ↓
21	Cholesterola	C_27_H_46_O	HMDB0000067	2.90E-02	2.54E-01	1.56	0.78 ↓
22	Phosphatidylethanolamine lyso alkenyl 18:1	C_23_H_46_NO_6_P	–	3.10E-02	2.54E-01	1.55	1.81 ↑
23	1-Aminocyclobutane carboxylic acid	C_5_H_9_NO_2_	–	3.14E-02	2.54E-01	1.55	0.56 ↓
24	Cystathionine	C_7_H_14_N_2_O_4_S	HMDB0000099	3.90E-02	3.01E-01	1.49	0.72 ↓
25	Proline	C_5_H_9_NO_2_	HMDB0000162	4.04E-02	3.01E-01	1.48	0.80 ↓
26	Lactic acid	C_3_H_6_O_3_	HMDB0000190	4.66E-02	3.22E-01	1.45	0.81 ↓
27	Acetylcholine chloride	C_7_H_16_ClNO_2_	HMDB0000895	4.92E-02	3.22E-01	1.43	0.71 ↓
28	Phosphatidylinositol 18:0–20:4	C_47_H_83_O_13_P	HMDB0009815	5.00E-02	3.22E-01	1.43	1.49 ↑

^a^: The *P* value was obtained by one-way ANOVA

^b^: The *P* value was adjusted by Benjamini-Hochberg method with the p.adjust function in R project

^c^: *VIP* variable importance in projection in the OPLS-DA model

^d^: *FC* Fold-change (HFSS/LFSS), values > 1 and < 1 indicate higher and lower levels in HFSS hens, respectively. Arrows indicate up- and down regulation of the compounds in HFSS individuals

To investigate the relationship among DEMs, a heatmap of the Pearson correlations among the 28 metabolites was created, which presented several metabolite clusters (Fig. 4c). In particular, seven metabolites that included two monoacylglycerols (monooctadecanoylglycerol and monohexadecanoylglycerol), four fatty acids (namely, arachidonic acid (C20:4n6); linoleic acid (C18:2n6); oleic acid (C18:1n9) and hexadecanoic acid (C16)) and a by-product of lipid peroxidation — (2E)-hexenal, exhibited significant correlations among themselves. Moreover, the differentially expressed compounds were subjected to pathway enrichment analysis to elucidate the mechanisms underlying the metabolic pathway changes in sperm storage (Additional file 6: Table S4). The results showed that the pathway related to the biosynthesis of unsaturated fatty acids was significantly enriched (FDR < 0.05, Fig. 4d).

### Components of carbohydrates and antioxidants in SSTs

**Fig. 5:**
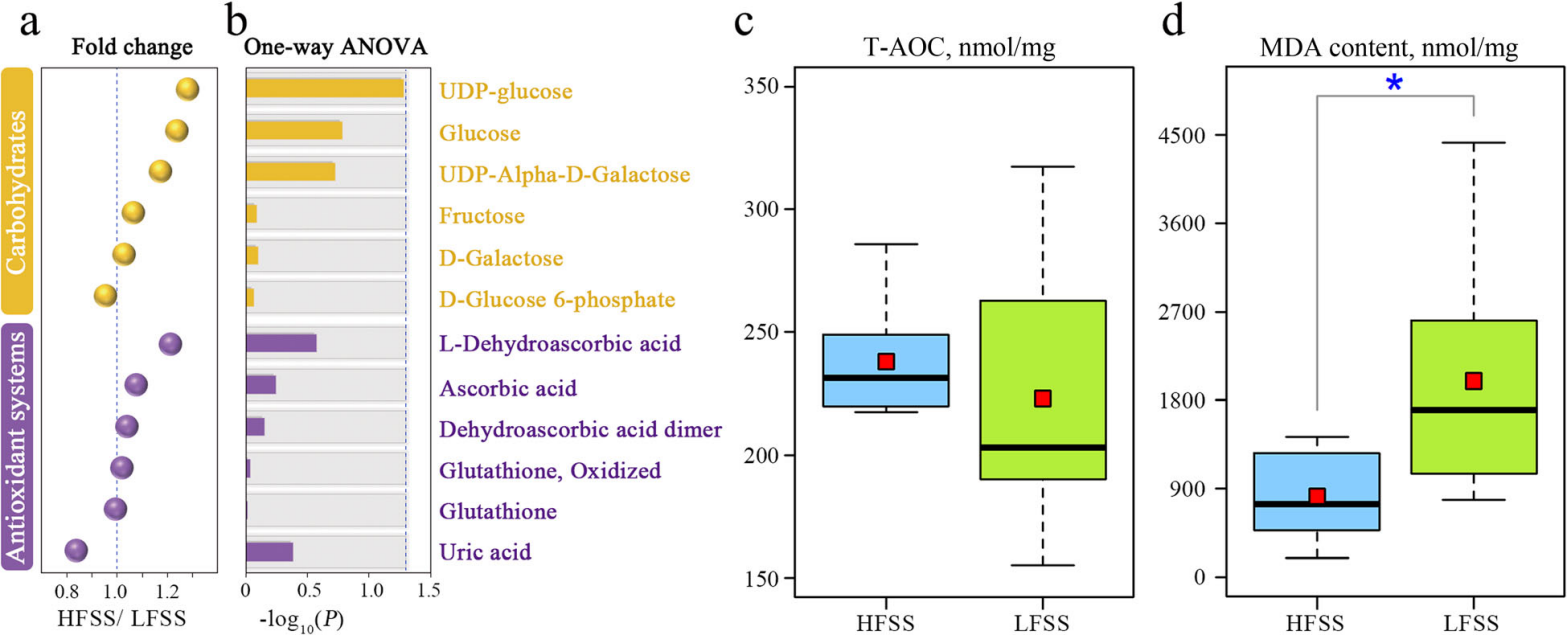
The differences in carbohydrate content and total antioxidant capacity (T-AOC) between the HFSS and LFSS groups. **a**-**b** Comparisons of carbohydrates, antioxidants and their intermediates between HFSS and LFSS. For **b**, the blue dashed line represents the *P* = 0.05. **c**-**d** The differences in the T-AOC and malondialdehyde (MDA) content between HFSS and LFSS. * indicated the *P* < 0.05

Given that carbohydrates may act as a possible energy source for sperm, and sperm membranes must be protected by a highly efficient antioxidant system to prevent peroxidative damage during prolonged storage. We analyzed the component of carbohydrates and non-enzymatic antioxidants for UVJ mucosal tissue, and detected three common monosaccharides: glucose, fructose and galactose, and their intermediates. The water-soluble antioxidants (including ascorbic acid, glutathione and uric acid) and their oxidation states were observed in the UVJ. Although the content of monosaccharides and antioxidants (except uric acid) was lower in the LFSS group than in the HFSS group (Fig. 5a), no significant differences were observed between the HFSS and LFSS groups (Fig. 5b). These results suggested that the contents of carbohydrates and antioxidants were not the critical factors leading to the differences in sperm storage ability among individuals.

### The T-AOC and lipid peroxidation level

Then, the UVJ tissues of both groups were used for detecting the T-AOC. The result corroborated the metabolomic results that the T-AOC was not significantly different between the two groups (Fig. 5c). As high levels of monoacylglycerols, fatty acids and (2E)-hexenal were observed in the LFSS groups, we speculated that lipid metabolism may have detrimental effects on sperm storage. To further confirm whether there was a difference in lipid peroxidation between the HFSS and LFSS groups, we determined the concentration of the final product of lipid peroxidation — MDA between the HFSS and LFSS groups. Our results showed that samples of UVJ mucosal tissue from the LFSS birds produced large amounts of MDA which was 2.4-fold greater than that produced by samples from the HFSS groups (Fig. 5d).

## Discussion

Successful fertilization requires viable spermatozoa and eggs to meet. The maintenance of fertility for long periods after copulation or insemination is widespread throughout the avian species. In the present study, we observed that the duration of sperm storage within the female RT was highly varied among hens. Based on the global metabolome analysis and multivariate statistics performed herein, we observed obvious differences in the metabolite composition in SSTs between the LFSS and HFSS hens. We propose that the long-term storage of sperm and the maintenance of their function in the female RT require an adequate microenvironment. Despite the highly organized lines of defense against peroxidation present in SSTs, it is noteworthy that the lipids, fatty acids and lipid peroxidative products were higher in the LFSS hens than in the HFSS individuals. The overabundance of fatty acids secreted from SSTs that alter SST lumen physiology caused by lipid peroxidation is an important factor explaining sperm storage limitations in chickens.

FSS is pervasive among organisms with internal fertilization, including mammals [44, 45], invertebrates [46, 47], reptiles [48, 49], fishes [50, 51] and amphibians [52]. A basic question is how to quantify this trait. The usual criteria for evaluating that a species stores sperm range from simply finding sperm within a female RT to more thorough determinations of both the female physiology and the sperm viability [3]. Assessing the capacity of FSS by a series of fertile eggs has been broadly applied in avians [10, 11, 53–55]. This idea will also be valuable to other oviparous animals, such as mosquitoes [46]. Given that most studies have suggested that genetics has moderate effects on FSS, we estimated the *h*^*2*^ of FE and FDD. The *h*^*2*^ for the two traits were moderate, and higher genetic correlations of these traits between the two experimental periods were obtained, and these results were comparable to those obtained in previous reports [10, 54, 56]. The results indicated that the criteria we used to assess the characteristics of FSS were applicable and suitable.

Apart from determining a suitable index to evaluate the FSS, more important questions are how hens prolong sperm storage and why the duration of FSS was distinct among individuals. Since Van Drimmelen [57] was first found that a large number of spermatozoa were stored in the mucosal crypts of chicken oviducts, sperm storage in the domestic fowl has been studied extensively at the morphological level [13–17]. The biological basis of sustained fertility in female birds is their capacity for keeping sperm activity in SSTs, which occurs widely across all groups of avian species [58]. Bakst et al. [19] suggested that the difference in the duration of fertility between chickens (2~3 weeks) and turkeys (10~15 weeks) is, in part, due to the increased number of SSTs and the resultant increase in sperm storage ability of the turkeys compared with that of chickens. Thus, we performed histological analysis of UVJ tissue and observed a significant difference in the density of SSTs between the HFSS and LFSS hens. A similar situation was reported by Adetula et al. [59], who found more SSTs were embedded in the HFSS hens compared with LFSS hens.

With the exception of the number of SSTs, a series of studies have shown that SSTs have the capacity to up-regulate or down-regulate sperm viability and motility through their secretion [24, 29], and hence, affect the duration of FSS. Sasanami et al. [22] found that the addition of UVJ extracts greatly extended the avian spermatozoa lifespan *in vitro*. That is, SSTs provide suitable and adequate chemical and biochemical compounds to sustain the viability and functional state of sperm in the female RT. Although extensive investigation concerning the condition and function of SSTs in birds has been performed since their discovery in the 1960s using ultrastructural analysis [25–27], few studies have been directed toward characterizing a precise metabolism and explaining the mechanisms involved in the prolonged FSS due to a lack of suitable approaches.

Inspired by the analysis of the female RT in human infertility and subfertility [60], we performed global metabolomics on the UVJ tissue of hens for obtaining valuable information about the metabolism within SST cells. The most prominent aspects of the SST metabolic network are glutathione metabolism and galactose metabolism. Glutathione is crucial for the antioxidant defense of cellular events [61]. As reviewed by Bréque et al. [33], and a complex antioxidant system including glutathione is present in SSTs to prevent sperm damage caused by peroxidation during storage. With respect to galactose metabolism, which plays an established role in energy delivery [62]. A considerable number of *in vitro* experiments has revealed that energy substrates, such as glucose and fructose added to diluents can prolong sperm viability and activity [63–65]. This indicated that high energy metabolic and antioxidant defense activities in the SST cells may be two of the factors responsible for long-term sperm retention or maintenance in the female RT. While our data showed that there was no significant difference in the content of monosaccharides and antioxidants and the T-AOC between the HFSS and LFSS groups, indicating that the level of carbohydrates and antioxidant capacity were not the critical factors leading to the differences in sperm storage ability among individuals.

For the detection of substances that are significantly associated with the duration of FSS, we performed multivariate statistical analysis and identified 28 components that exhibited different concentrations between hens with distinct sperm storage abilities. These DEMs were significantly enriched in the biosynthesis of unsaturated fatty acids pathway, indicating that lipid metabolism is important for FSS. Previous studies have consistently shown that large amounts of lipid components are present in SST cells [25–28]. Recently, Huang et al. [29] extracted the total lipids from homogenized mucosal tissues of UVJ, and identified that two unsaturated fatty acids (linoleic acid and oleic acid) and three saturated fatty acids (hexadecanoic acid, stearic acid and myristic acid) were the predominant fatty acids present in the chicken UVJ mucosa. Furthermore, Huang et al. [29] suggested sperm viability was improved by oleic acid and linolic acid in *in vitro* study. In our study, however, we found that the content of lipids and four fatty acids, arachidonic acid, linoleic acid, oleic acid and hexadecanoic acid, in the LFSS individuals were significantly higher than that in the HFSS hens. Arachidonic acid and linoleic acid are two *ω*-6 polyunsaturated fatty acids (PUFAs), and oleic acid is a part of the *ω*-9 monounsaturated fatty acid family (MUFAs). In a previous study, infertile males were found to have increaed blood serum and seminal plasma levels of *ω*-6 PUFAs, which resulted in decreased sperm motility and an increased malformation rate [66]. Conquer et al. [67] reported that seminal plasma from asthenozoospermic individuals have higher levels of oleic acid and MUFAs than those from normozoospermic males. More importantly, the superabundance of unsaturated fatty acids would increase the availability of substrate for lipid peroxidation. In the present study, the α-β unsaturated aldehyde — (2E)-hexenal was significantly higher in LFSS chickens. (2E)-hexenal is released during lipid peroxidation and exhibits cytotoxic activity [68]. Early reports by Jones et al. [69] confirmed that exogenous fatty acid peroxides have powerful spermicidal properties. Thus, we speculated that the superabundance of fatty acids secreted by SSTs has detrimental effects on FSS in chickens, and an intriguing possibility is that fatty acids are involved in oxidative degradation and generate some toxic products, although a highly efficient antioxidant system is present in the female RT. These lipid peroxides may cause irreversible damage to resident sperm, resulting in short-term sperm retention and decreased fertility.

To confirm our conjecture, we further evaluated the content of MDA in the UVJ mucosal tissues given that MDA is the principal and most studied biological marker for lipid peroxidation [70]. Our results showed an increased MDA content in LFSS hens compared to that in HFSS hens. Because of their relative stability and high reactivity, MDA can easily diffuse across membranes and has a high capability of reaction with multiple biomolecules (such as proteins or DNA) forming adducts [71]. Several studies in chickens have revealed that the formation of MDA is positively correlated with a dramatic reduction in fertilizing capabilities [72, 73]. A similar observation was described in turkeys [74]. We noticed a negative association between FSS ability and lipid metabolism, but UVJ tissue contains not only SSTs but also other tissue elements, further experiments should be conducted to explore the effect of lipid peroxidation on spermatozoa in the future.

## Conclusions

The biological basis of sustained fertility in chickens is their capacity to store sperm in SSTs. The results we reported here have demonstrated that the FSS differences are attributable, in part, to the physiological differences. Chickens with longer fertility duration have a greater density of SSTs than the chickens with short fertility duration. Females with more SSTs had the ability to store more sperm. More importantly, it seems likely that SSTs contribute to sustained sperm storage by secretions that alter the female reproductive physiology. Our study showed that the superabundance of fatty acids released into the SST lumen renders them vulnerable to lipid peroxidation and subsequently results in many toxic biochemical compounds, which may cause irreversible damage to resident sperm, resulting in short sperm storage ability. These findings suggest a detrimental physiological role for fatty acids in FSS. The increased knowledge of the functions of SST secretions will add a new dimension to sustain fertility and breeding behavior.

## Supplementary information

**Additional file 1: Figure S1.** The fertility and hen-day laying rate during the two test periods. Period 1 represents the first experimental period from 245 to 256 days of age (12 days), and period 2 represents the second experimental period from 378 to 392 days of age (15 days).

**Additional file 2: Table S1.** Descriptive statistics of duration of fertility and hatching egg production (HEP) in the two test periods.

**Additional file 3: Figure S2.** Histogram of individual hatching egg production (HEP), fertility and fertility duration days (FDDs) during the two test-experiment periods. Period 1 represents the first experimental period from 245 to 256 days of age (12 days), and period 2 represents the second experimental period from 378 to 392 days of age (15 days). If individual HEP was less than 3 (period 1) and 4 (period 2), the corresponding fertility and FDD were considered as miss values.

**Additional file 4: Table S2.** Physiological parameters and duration of fertility of hens that used for metabolomics study.

**Additional file 5: Table S3.** Metabolome profile of the UVJ tissue.

**Additional file 6: Table S4.** Pathway analysis of differentially expressed metabolites.

## Data Availability

All data generated or analyzed during this study are included in this published article and its supplementary information files.

## Abbreviations

DEMs: Differentially expressed metabolites
FDDs: Fertility duration days
FE: Fertility
FSS: Female sperm storage
GC-MS: Gas chromatography mass spectroscopy
*h*^*2*^: Heritability
HEP: Hatching egg production
HFSS: Higher sperm storage ability
LFSS: Lower sperm storage ability
MDA: Malondialdehyde
MUFAs: Monounsaturated fatty acids
PCA: Principal component analysis
PUFAs: Polyunsaturated fatty acids
RT: Reproductive tract
SSTs: Sperm storage tubules
T-AOC: Total antioxidant capacity
UPLC-MS: Ultrahigh-performance liquid chromatography mass spectrometry
UVJ: Utero-vaginal junction
VIP: Variable importance in projection
