# Supplementary figures and images for: Detrimental effects of excessive fatty acid secretion on female sperm storage in chickens
Source: J Anim Sci Biotechnol. 2020 Apr 2;11:26. doi: 10.1186/s40104-020-0432-8 (PMC7114795; doi:10.1186/s40104-020-0432-8)

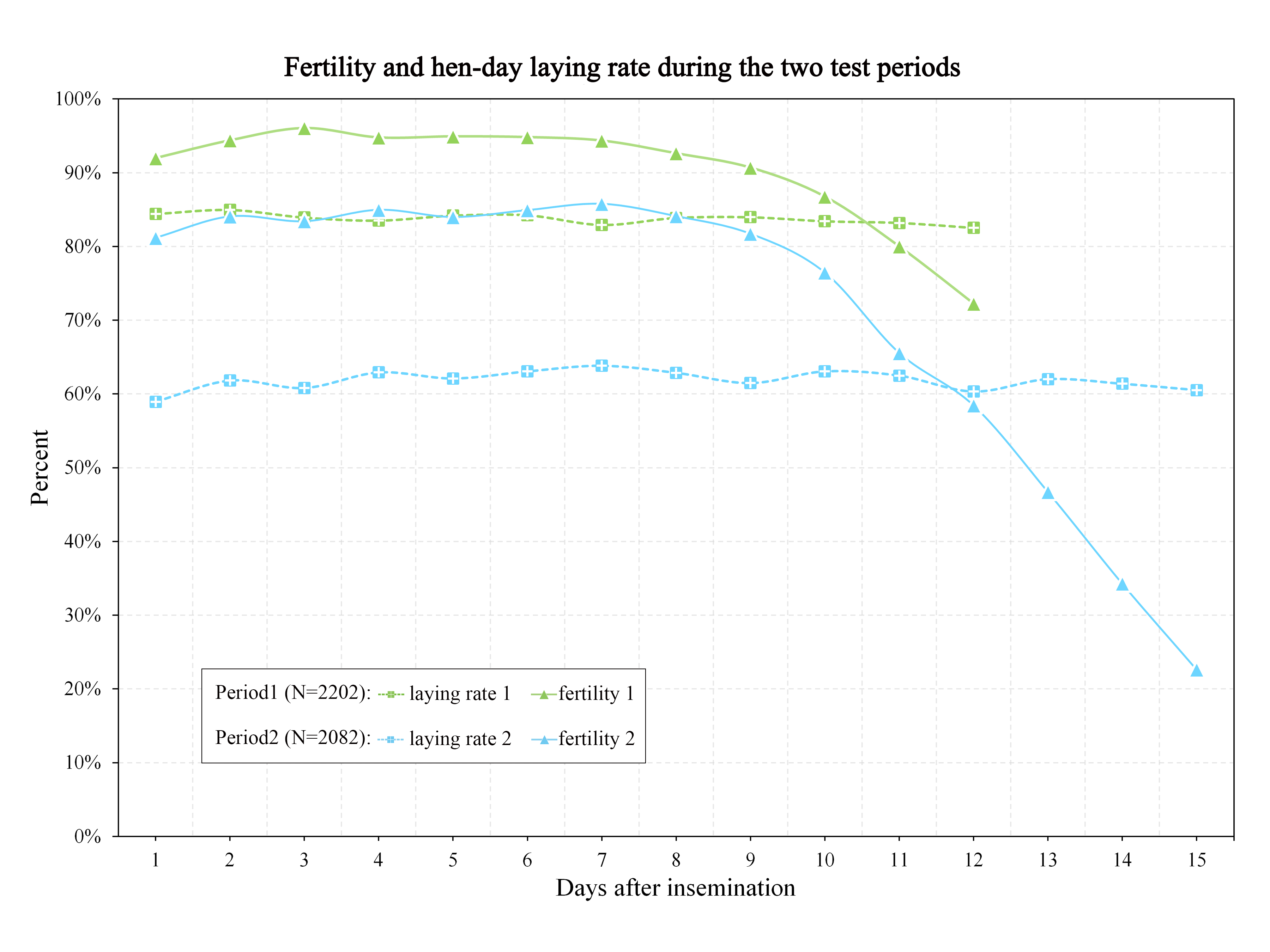

Supplement: Supplementary file 1 — Additional file 1: Figure S1. The fertility and hen-day laying rate during the two test periods. Period 1 represents the first experimental period from 245 to 256 days of age (12 days), and period 2 represents the second experimental period from 378 to 392 days of age (15 days). [file 40104_2020_432_MOESM1_ESM.tif]

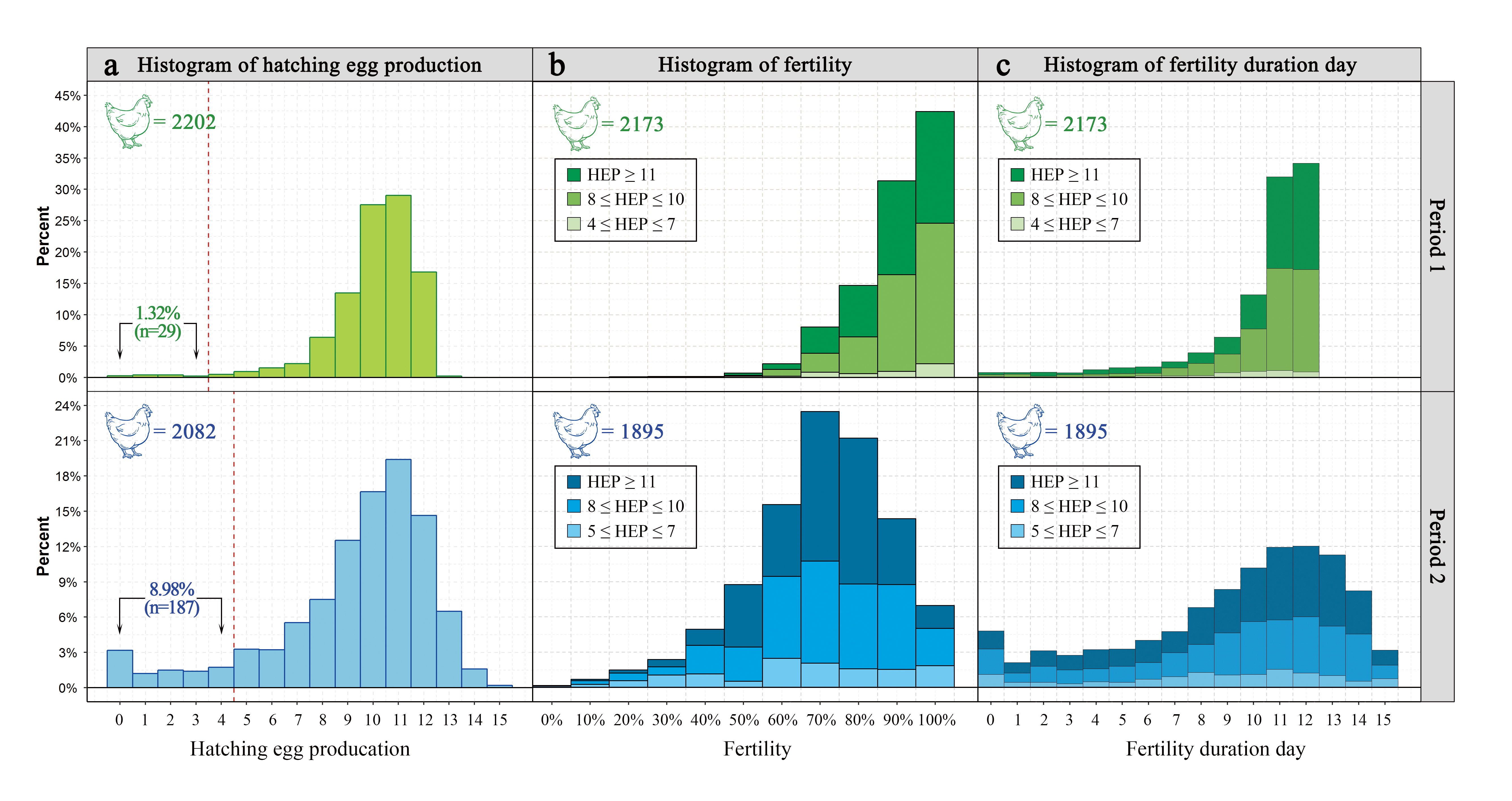

Supplement: Supplementary file 3 — Additional file 3: Figure S2. Histogram of individual hatching egg production (HEP), fertility and fertility duration days (FDDs) during the two test-experiment periods. Period 1 represents the first experimental period from 245 to 256 days of age (12 days), and period 2 represents the second experimental period from 378 to 392 days of age (15 days). If individual HEP was less than 3 (period 1) and 4 (period 2), the corresponding fertility and FDD were considered as miss values. [file 40104_2020_432_MOESM3_ESM.tif]
